# Growth Impairment and Neurodevelopmental Features in Kabuki Syndrome Type 2: A Case Report With a Novel *KDM6A* Frameshift Mutation

**DOI:** 10.1155/crpe/3013437

**Published:** 2026-11-04

**Authors:** Sayan Najafzade, Haneieh Honarmand, Mortaza Bonyadi, Mohammad Barzegar

**Affiliations:** ^1^ Animal Biology Department Faculty of Natural Sciences University of Tabriz Tabriz Iran tabrizu.ac.ir; ^2^ Center of Excellence for Biodiversity Faculty of Natural Sciences University of Tabriz Tabriz Iran tabrizu.ac.ir; ^3^ Pediatric Health Research Center Mardani Azari Children Hospital Tabriz University of Medical Sciences Tabriz Iran tbzmed.ac.ir

**Keywords:** c.1083_1084delCT, case report, *de novo* mutation, Kabuki syndrome, *KDM6A*

## Abstract

Kabuki syndrome (KS), a rare epigenetic disorder, arises from pathogenic variants in *KMT2D* (Kabuki syndrome Type 1 [KS1]; 70%–75% of the cases) or *KDM6A* (Kabuki syndrome Type 2 [KS2]; 5%–10% of the cases), exhibiting autosomal dominant and X‐linked dominant inheritance patterns, respectively. This study describes a 13‐year‐old girl with classic KS2 manifestations including intellectual disability, ADHD, and short stature (height 130 cm, < 3rd percentile), alongside characteristic dysmorphic features. Her clinical history revealed spontaneous atrial septal defect closure, failure to thrive, and delayed bone age. Whole‐exome sequencing uncovered a novel *KDM6A* frameshift variant (c.1083_1084delCT), confirmed as *de novo*, which introduces a premature termination codon in exon 12 (> 50 bp from splice junctions). Computational predictions suggest that the variant likely triggers nonsense‐mediated decay (NMD), resulting in haploinsufficiency. This predicted NMD‐mediated haploinsufficiency is consistent with the established loss‐of‐function disease mechanism of KDM6A‐associated KS2. KDM6A is located on the X chromosome, consistent with the X‐linked classification of this condition. Population studies—including analysis of our cohort of 500 ethnically matched individuals and published data from resources like gnomAD—demonstrate the absence of this variant in healthy populations, further supporting the rarity of the variant (PM2). These findings expand the known mutational spectrum of KS2 and suggest that NMD‐mediated haploinsufficiency may represent a key molecular mechanism. The case highlights the clinical distinctiveness of KS2, particularly its endocrine and growth‐related manifestations, and expands the mutational and phenotypic spectrum of KDM6A‐associated KS2.

## 1. Introduction

Kabuki syndrome (KS) is a rare genetic disorder characterized by five principal clinical manifestations: distinctive facial dysmorphism (most notably elongated palpebral fissures with lateral eversion of the eyelids), skeletal anomalies, dermatoglyphic abnormalities such as persistent fetal fingertip pads, mild‐to‐moderate intellectual disability (ID) and developmental delay, and impaired postnatal growth [1]. The syndrome exhibits substantial phenotypic variability, extending to structural abnormalities in cardiac, renal, genitourinary, and auditory systems; dental anomalies (e.g., hypodontia); hypotonia; feeding issues; hearing loss; microcephaly; and diverse multisystem involvements across immune, neurologic, ophthalmologic, gastrointestinal, and endocrine functions. Notable endocrine complications include hyperinsulinemic hypoglycemia, affecting 0.3%–4% of the cases [2, 3].

The syndrome arises from pathogenic variants in either of two genes: *KMT2D* (accounting for 70%–75% of the cases) or *KDM6A* (responsible for 5%–10% of the cases). Deleterious heterozygous variants in *KMT2D* underlie the autosomal dominant subtype designated as Kabuki syndrome Type 1 (KS1, MIM #147920), in contrast to heterozygous or hemizygous variants in *KDM6A*, which are linked to the X‐linked dominant form known as Kabuki syndrome Type 2 (KS2, MIM #300867) [4, 5].

Genetic testing in KS provides definitive molecular confirmation, helping to end prolonged diagnostic uncertainty and differentiate KS from clinically overlapping conditions. Early diagnosis enables targeted surveillance of cardiac, endocrine, immune, and developmental systems, helping to address common complications including feeding difficulties, recurrent infections, and developmental delays. Molecular confirmation further informs prognosis and allows accurate genetic counseling for families. In addition, identifying the underlying genetic variant may determine eligibility for emerging targeted therapies. Continued accumulation and systematic analysis of clinical phenotypes and mutation spectra are, therefore, essential to advance understanding of the disorder [6].

A novel *de novo* likely pathogenic variant (NM_001291415.2, c.1083_1084delCT, p.Tyr362fs) in the *KDM6A* gene was identified by whole‐exome sequencing (WES) in a 13‐year‐old girl exhibiting clinical features consistent with KS, as detailed in this report, thereby adding new insight into the genetic architecture and clinical variability of KS.

## 2. Materials and Methods

### 2.1. Case Analysis

A 13‐year‐old female patient from a nonconsanguineous family was referred to the Pediatric Neurology Division at Mardani Azari Children’s Hospital (East Azerbaijan Province, Iran) for evaluation of ID, attention deficit and hyperactivity disorder (ADHD), short stature, and characteristic dysmorphic features. Diagnostic evaluation included comprehensive pedigree analysis, detailed clinical assessment, laboratory investigations, and longitudinal follow‐up. Written informed consent was obtained from her legal guardians prior to study inclusion. The research protocol was approved by the Ethics Committee of the University of Tabriz (IR.TBZMED.REC.1404.384).

### 2.2. Genetic Analysis

#### 2.2.1. WES

WES was performed on the proband after obtaining informed consent from the legal guardian. Genomic DNA was extracted from peripheral blood and enriched using the Agilent SureSelect Human All Exon V7 kit. Sequencing was conducted on the Illumina NovaSeq 6000 platform (150 bp reads), aligned to GRCh38/hg38 and GRCh37/hg19 using CLC Genomics Workbench and BWA [7, 8]. Low‐quality reads (Qbase < 20) and duplicates were filtered using Picard and Trimmomatics (v0.39) [9]. Variant calling (SNVs/indels) was performed with GATK (v4.2.0.0) [10] and DeepVariant (v1.1.0) [11], followed by annotation using wANNOVAR [12–14].

Variants were filtered using an in‐house pipeline based on Sherloc and ACMG guidelines, excluding synonymous, deep intronic, and noncoding variants (except exon‐adjacent regions) [15]. Common variants (MAF > 0.05) were excluded. Pathogenicity was assessed using CADD (Phred ≥ 10) [16], MutationTaster [16], OMIM [17], Franklin, and VarSome [17]. Candidate variants were validated via Sanger sequencing. RNA secondary structure and protein impact were predicted using online tools [18, 19]. The NM_001291415.2 (c.1083_1084del) variant was analyzed using ExPASy Translate Tool and NMDEscPredictor, confirming that it introduces a premature stop codon and is predicted to be targeted by nonsense‐mediated mRNA decay [20, 21].

#### 2.2.2. Sanger Sequencing Validation of the Likely Pathogenic Variant

Sanger sequencing was performed on the proband, and both parents to confirm the likely pathogenic variant and to further evaluate its association with the disease. Sequencing was carried out using the ABI 3500 Genetic Analyzer (Applied Biosystems). For targeted amplification, custom primers were designed to flank the variant of interest: forward primer: 5′‐CAGCAGCAAAATCAGCCCAT‐3′, reverse primer: 5′‐TGCTGCAAGTGCAGAGGTAT‐3′.

During validation, genomic DNA from the proband and parents was amplified by PCR using Taq DNA polymerase with an annealing temperature of 59°C. The resulting PCR products were then subjected to Sanger sequencing to confirm the presence of the variant.

### 2.3. Control Population Analysis

To contextualize the rarity of the identified variant, we compared our patient’s WES data against an in‐house control cohort of 500 healthy individuals of matched Iranian–Azeri–Turkish ethnicity. This control group was specifically analyzed for this case report to establish the ethnic‐specific baseline allele frequency, as this variant has not been previously documented in this population.

## 3. Results

### 3.1. Case History

The patient was a 13‐year‐old girl with ID and short stature, referred to the outpatient neurology clinic for evaluation. She was born via normal vaginal delivery at 38 weeks’ gestation, with a birth weight of 3250 g, length of 49 cm, and head circumference of 34 cm. Her prenatal and perinatal periods were unremarkable.

The patient had an atrial septal defect (ASD), which closed spontaneously without intervention. She experienced feeding difficulties since infancy, leading to failure to thrive. Due to her short stature, a bone age evaluation at 5 years of age revealed a 2‐year delay compared to her chronological age, raising suspicion of an endocrine etiology.

Blood tests confirmed growth hormone deficiency (GHD), and she was started on growth hormone therapy. However, her final height remained below the normal range. She achieved developmental milestones with significant delays—independent walking at 4 years and speech at 4 years.

At age 13 years, psychometric evaluations showed ID with ADHD. No history of seizures has been reported. Her growth parameters at this age were height 130 cm (below the 3rd percentile), weight 28 kg (below the 3rd percentile), and microcephaly with head circumference of 50.5 cm (significantly below the normal range for age).

Neurological examination reveals dysmorphic facial features including large auricles (with normal hearing), sparse lateral eyebrows, long palpebral fissures, a broad and depressed nasal tip, and a high‐arched palate. She also has brachydactyly, persistent fetal fingertip pads (without hand involvement), and joint hyperlaxity. These findings suggest KS2 as a possible diagnosis.

### 3.2. WES Data

The proband was referred to the genetic laboratory for further evaluation using WES. A heterozygous CT deletion at position 1083 (NM_001291415.2, c.1083_1084delCT, p.Tyr362fs) in exon 12 of the *KDM6A* gene was identified (Figure 1A). Sanger sequencing results revealed that the parents had normal genotypes at this locus, whereas the proband was heterozygous for the variant (Figure 1B). Sanger sequencing confirmed the *de novo* occurrence of the variant, which is consistent with the known X‐linked dominant inheritance pattern of KDM6A‐associated KS2.

**FIGURE 1:**
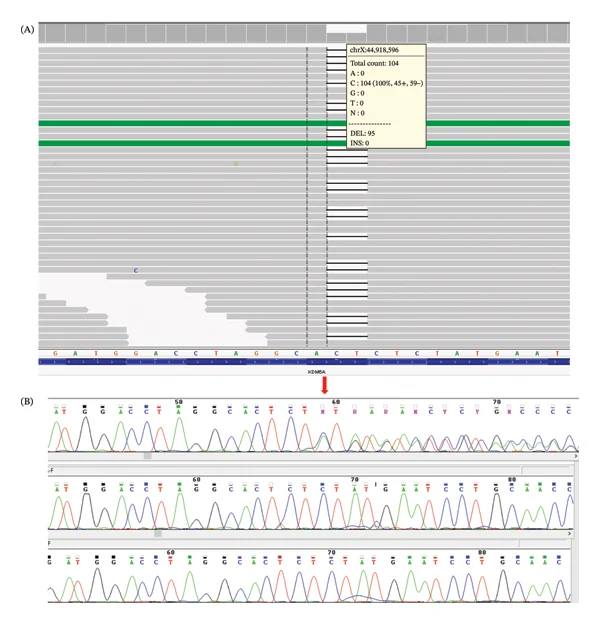
Whole‐exome sequencing and Sanger sequencing results in the proband and her parents. The likely pathogenic variant in KDM6A gene (c.1083_1084) is visualized in the IGV figure of the proband’s BAM file (A). The variant is present in the proband in a heterozygous state and is absent in both parents, consistent with a de novo occurrence (B).

### 3.3. Bioinformatics Analysis

ExPASy Translate Tool analysis (https://web.expasy.org/translate/) showed that the patient’s variant (NM_001291415.2, c.1083_1084delCT) is a frameshift variant that introduces a premature termination codon (PTC), predicted to result in a truncated protein. NMDEscPredictor (https://nmdprediction.shinyapps.io/nmdescpredictor/) predicted that this variant is likely targeted by nonsense‐mediated mRNA decay (degradation probability: high/NMD‐sensitive), suggesting the mutant mRNA is expected to be degraded, consistent with a predicted loss‐of‐function mechanism (Figure 2). Conservation analysis indicated that the affected region is conserved across species (Figure 3).

**FIGURE 2:**
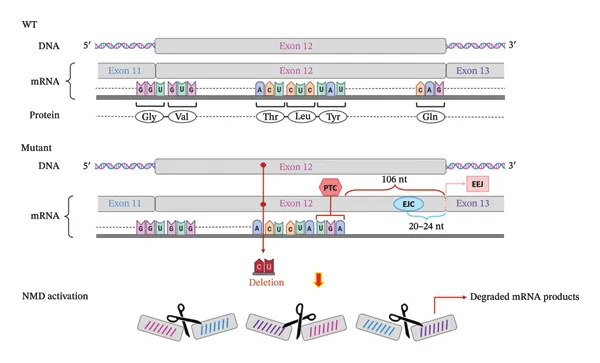
Predicted impact of the KDM6A c.1083_1084del frameshift on transcript stability. The deletion causes a frameshift in the coding sequence and introduces a premature termination codon (PTC) upstream of the final exon–exon junction, leaving downstream exon junction complexes (EJCs) on the mRNA. This configuration is expected to trigger nonsense‐mediated mRNA decay (NMD), leading to degradation of the mutant transcript and loss of KDM6A function.

**FIGURE 3:**
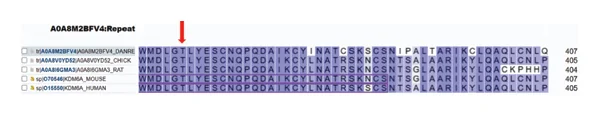
Multiple sequence alignment of KDM6A orthologs from human, mouse, rat, chicken, and zebrafish demonstrates high evolutionary conservation of the region encompassing Tyr362. The identified frameshift variant (c.1083_1084del) is predicted to disrupt this conserved region, highlighting its functional importance and supporting a loss‐of‐function mechanism for KDM6A.

In addition, we performed exploratory RNA secondary structure analysis using the RNAsnp web‐based tool, which revealed no significant difference between the wild‐type and mutant transcripts (*p* > 0.05). However, as this finding is based on in silico prediction rather than experimental validation, it should be interpreted with caution and does not constitute functional evidence of pathogenicity. Similarly, protein structural modeling was explored but is not presented here, as computational predictions alone do not provide confirmatory evidence for variant pathogenicity under current variant interpretation guidelines.

### 3.4. Population Screening

This variant was absent from 500 healthy individuals from the Iranian–Azeri–Turkish ethnic group and from public population databases, including the 1000 Genomes Project and gnomAD, supporting its rarity (PM2).

This variant is classified as likely pathogenic based on multiple ACMG criteria: the patient’s characteristic clinical presentation (PP4), confirmed de novo occurrence with verified parentage and no family history (PS2), and absence from population databases (PM2). Furthermore, the frameshift variant in exon 12 is predicted to introduce a PTC, triggering nonsense‐mediated mRNA decay and resulting in haploinsufficiency of the KDM6A protein (PVS1). Loss of function is a well‐established disease mechanism for KDM6A‐associated KS2. Notably, KDM6A has multiple alternatively spliced transcripts; the variant described herein affects the major transcript (NM_001291415) and is predicted to result in loss of function across all biologically relevant isoforms. Consistent with this interpretation, 79 pathogenic null variants have been reported across 24 exons in ClinVar, including several within the same exon. Collectively, these findings substantiate the classification of this variant as likely pathogenic.

## 4. Discussion

KS, first described by Niikawa and Kuroki in 1981, is a rare congenital disorder primarily caused by pathogenic variants in the *KMT2D* or *KDM6A* genes. Two molecular subtypes are recognized: Type 1 (KS1), associated with *KMT2D* mutations, and Type 2 (KS2), resulting from pathogenic variants in *KDM6A*, an X‐linked gene located at Xp11 that largely escapes X‐chromosome inactivation. *KDM6A* encodes a 1401‐amino‐acid protein featuring two key functional domains. Its catalytic domain, spanning exons 18–21, functions as a histone demethylase that selectively erases mono‐, di‐, and trimethyl marks from lysine 27 on histone H3 (H3K27). This enzymatic activity is crucial for modulating tissue‐specific gene expression, with significant involvement in developmental pathways and cell cycle regulation. The second functional domain of KDM6A contributes to chromatin remodeling via its interaction with the SWI/SNF (switch/sucrose non‐fermentable) complex, which includes the transcriptional activator BRG1 [22]. Recent work by Chi et al. revealed that *KDM6A* variants in KS impair histone demethylase activity through multiple mechanisms, including disruptions in protein integrity, local chromatin environment, molecular interactions, and protein dynamics [23]. The pronounced postnatal growth failure frequently observed in KS, characterized by severe height deficits and delayed bone maturation, may stem from the disrupted interplay between KDM6A and the SWI/SNF chromatin remodeling complex, which is critical for skeletal development [3, 22, 24, 25]. Furthermore, the transition from normal birth head circumference to acquired microcephaly highlights the gene’s essential role in postnatal neurodevelopment [26]. Preclinical models corroborate this, demonstrating that KDM6A promotes neuronal differentiation by demethylating H3K27me3 at promoters of key proneural genes like *Ngn3*, thereby ensuring proper cortical organization [25, 27].

Here, we report a novel *de novo* frameshift mutation (NM_001291415.2, c.1083_1084delCT, p.Tyr362fs) in exon 12 of the *KDM6A* gene, which generates a PTC, in a 13‐year‐old girl. Given the mutation’s location (not in the terminal exon or within 50 nucleotides of the penultimate exon’s 3′ end), the transcript is predicted to undergo NMD, resulting in *KDM6A* haploinsufficiency. The patient’s multisystem presentation provides a clinically instructive example of *KDM6A*‐related KS, demonstrating both classic and atypical features of this epigenetic disorder. Her growth pattern exemplifies the complex endocrine manifestations seen in KS, in which GHD occurs in approximately 50% of the cases. Her suboptimal response to GH therapy is consistent with previous observations [28].

The patient’s neurodevelopmental trajectory—achieving independent walking and speech at 4 years, followed by ADHD and ID diagnosis—aligns with the 82% prevalence of developmental delays in molecularly confirmed KS cases [29]. However, the absence of seizures contrasts with the 28%–38% epilepsy risk reported in larger cohorts [30], potentially indicating milder neurological involvement in some *KDM6A* variants. Her distinctive facial features (long palpebral fissures, sparse lateral eyebrows, and broad nasal tip) satisfy clinical diagnostic criteria for KS [31], while the persistence of fetal fingertip pads and joint hyperlaxity underscore *KDM6A*’s role in connective tissue homeostasis, likely through modulation of extracellular matrix genes [32]. The spontaneous closure of her ASD, observed in 30%–40% of KS cardiac anomalies [33], illustrates the variable penetrance of cardiovascular manifestations.

Finally, the case underscores three key clinical implications: First, the development of microcephaly postnatally suggests *KDM6A* mutations may affect ongoing neurogenesis rather than just early brain development. Second, the limited efficacy of GH therapy indicates the need for multidisciplinary growth management in KS. Third, the absence of seizures does not exclude KS2 diagnosis, expanding the phenotypic spectrum of *KDM6A*‐related disorders. Future studies should investigate whether specific *KDM6A* mutation types correlate with neurological severity and how *KDM6A* interacts with growth plate signaling pathways.

Functional studies, such as studies using knock‐in/knock‐out mice models and biochemical assays (e.g., chromatin immunoprecipitation sequencing to assess histone methylation changes), can delineate the causal impact of *KDM6A* variants on critical processes like neurogenesis and metabolic regulation.

According to the ACMG guidelines, this variant is classified as likely pathogenic. Sanger sequencing confirmed a *de novo KDM6A* mutation in the patient, which was absent in both parents.

## 5. Conclusion

In this study, we identified a novel frameshift variant (NM_001291415.2: c.1083_1084delCT) in the *KDM6A* gene in a 13‐year‐old proband with KS2. Sanger sequencing confirmed the *de novo* occurrence of the variant, which was absent in both parents and in population databases. Bioinformatics analysis predicted that the variant introduces a PTC, likely resulting in nonsense‐mediated mRNA decay and loss of function, consistent with the established disease mechanism for KDM6A‐associated KS2. This report expands the mutational spectrum of *KDM6A* and provides further evidence supporting the role of loss‐of‐function variants in the pathogenesis of this condition. These findings may contribute to genetic counseling and diagnostic approaches for families affected by KS2.

## Author Contributions

Sayan Najafzade: investigation, formal analysis, and writing–original draft. Haneieh Honarmand: writing–original draft. Mortaza Bonyadi: conceptualization, supervision, and validation. Mohammad Barzegar: conceptualization and supervision.

## Funding

This research did not receive any specific grant from funding agencies in the public, commercial, or not‐for‐profit sectors.

## Ethics Statement

This study was performed in line with the principles of the Declaration of Helsinki. Approval was granted by the Ethics Committee of the Medical University of Tabriz (IR.TBZMED.REC.1404.384).

## Consent

All the patients allowed personal data processing, and informed consent was obtained from all individual participants included in the study.

## Conflicts of Interest

The authors declare no conflicts of interest.

## Data Availability

The data that support the findings of this study are available from the corresponding authors upon reasonable request.
